# Improving Patients’ Medication Adherence and Outcomes in Nonhospital Settings Through eHealth: Systematic Review of Randomized Controlled Trials

**DOI:** 10.2196/17015

**Published:** 2020-08-20

**Authors:** Zoie SY Wong, Braylien Siy, Katharina Da Silva Lopes, Andrew Georgiou

**Affiliations:** 1 Graduate School of Public Health St. Luke's International University Tokyo Japan; 2 Australian Institute of Health Innovation Macquarie University Sydney Australia

**Keywords:** eHealth, self-administered drug, self-management, medication adherence, nonhospital settings, randomized controlled trial

## Abstract

**Background:**

Electronic health (eHealth) refers to the use of information and communication technologies for health. It plays an increasingly important role in patients’ medication management.

**Objective:**

To assess evidence on (1) whether eHealth for patients’ medication management can improve drug adherence and health outcomes in nonhospital settings and (2) which eHealth functions are commonly used and are effective in improving drug adherence.

**Methods:**

We searched for randomized controlled trials (RCTs) on PubMed, MEDLINE, CINAHL, EMBASE, EmCare, ProQuest, Scopus, Web of Science, ScienceDirect, and IEEE Xplore, in addition to other published sources between 2000 and 2018. We evaluated the studies against the primary outcome measure of medication adherence and multiple secondary health care outcome measures relating to adverse events, quality of life, patient satisfaction, and health expenditure. The quality of the studies included was assessed using the Cochrane Collaboration’s Risk of Bias (RoB) tool.

**Results:**

Our initial search yielded 9909 records, and 24 studies met the selection criteria. Of these, 13 indicated improvement in medication adherence at the significance level of *P*<.1 and 2 indicated an improved quality of life at the significance level of *P*<.01. The top 3 functions that were employed included mechanisms to communicate with caregivers, monitoring health features, and reminders and alerts. eHealth functions of providing information and education, and dispensing treatment and administration support tended to favor improved medication adherence outcomes (Fisher exact test, *P*=.02). There were differences in the characteristics of the study population, intervention design, functionality provided, reporting adherence, and outcome measures among the included studies. RoB assessment items, including blinding of outcome assessment and method for allocation concealment, were not explicitly reported in a large number of studies.

**Conclusions:**

All the studies included were designed for patient home-based care application and provided a mechanism to communicate with caregivers. A targeted study population such as older patients should be considered to maximize the public health impact on the self-management of diseases.

**Trial Registration:**

International Prospective Register of Systematic Reviews (PROSPERO) CRD42018096627; https://www.crd.york.ac.uk/prospero/display_record.php?RecordID=96627

## Introduction

Integrating electronic health (eHealth) into medication prescription, dispensing, and administration processes is a promising step in the direction of achieving better medication safety, treatment, and health outcomes [1]. Health technology that supports patients’ medication management can be integrated into different media including mobile health (mHealth) [2], telehealth [3], SMS, and wearable devices [1]. Offering a range of functionalities such as remote consultation and monitoring essential health indicators [1], eHealth plays essential roles in informing, educating, connecting, monitoring, and motivating patients [4].

Noncompliance with medication regimes on the part of patients is common, particularly among those who suffer from chronic diseases such as diabetes, hypertension [5], and cardiovascular conditions [6]. Failure to adhere to medication regimes can lead to poor health outcomes and increased health care costs [7]. Studying improvements in medication adherence has become an important area of focus in eHealth [6,8]. While evaluating eHealth, it is also common to consider several essential health care outcomes [9-12]. The recent literature has examined the impact of eHealth on patient safety [9,13], quality of life [14], and satisfaction [15] as well as health care spending [16].

This study is a systematic review that investigates how eHealth impacts the outcomes of patients’ self-medication management. Based on the definition offered by the World Health Organization, this study defines eHealth [17] as referring to advancements in information and communication technologies that support care delivery and patient health management [18]. Instead of prescribing electronic medication in hospitals [9,13], we are interested in how eHealth contributes to the change in medication-taking behaviors in the nonacute disease management and recovery phases. We focus on drug-taking events in nonhospital and nonacute settings. These settings include home care, long-term care for older people, rehabilitation care, and outpatient facilities [19,20].

Prior studies have incorporated various methods of evaluation, such as rating systems and scales, user testing, and content analysis, to assess eHealth targeting for medication adherence [21]. This study focuses only on randomized controlled trials (RCTs), as this approach is the gold standard for evaluating digital medical intervention studies [22,23]. Drawing upon the current body of RCT studies, this review aims to assess the best available evidence on how eHealth interventions for self-management of medication improves drug adherence and health care outcomes. At the same time it characterizes the eHealth functions that are most commonly incorporated and those that favor improved medication adherence. In doing so, the study will contribute to the design, application, and sustainable development of eHealth in patient self-medication management.

## Methods

### Literature Search

The search was carried out in August 2018. To ensure exhaustive search results, cross-sectional databases in the fields of medicine, nursing care, public health, science, engineering, and social science were covered. The following databases were searched from 2000 to August 2018: PubMed, MEDLINE, CINAHL, EMBASE, EmCare, ProQuest, Scopus, Web of Science, ScienceDirect, and IEEE Xplore. We also included the Cochrane Library and gray literature sources, including Google Scholar and Open Access Theses and Dissertations. The snowball method was used to manually search citations within the studies included. We also hand-searched all the RCTs from the Journal of Medical Internet Research (JMIR) journals. Owing to the variation in terminology used to describe the topic of interest, we employed a broad, inclusive search strategy that covered the concepts of medication administration, eHealth, and nonhospital settings. Considering the appropriate Medical Subject Headings (MeSH) terms, we developed a set of master search terms that were applied to electronic data sets (enclosed in the Multimedia Appendix 1).

The inclusion criteria are presented as follows. We included all RCT studies that examined the effect of an eHealth intervention involving medication administration in a nonhospital setting. The periods for intervention needed to be at least six months. eHealth covers a broad range of mHealth, digital health, telehealth, electronic messaging, and electronic reminder interventions. Purely telephone-based outreach was not considered as health technology. We included studies in which participants needed to take medication regularly under nonacute settings. Only studies that focused solely on oral drug administration were selected. If the study did not specify a medication route or the intervention was administrated through a variety of routes (eg, [24]), it was not included. Studies were evaluated only when they reported, either directly or indirectly, on drug medication adherence, health care outcomes in adverse drug events, patient satisfaction, costs, or quality of life. Cluster and pragmatic RCTs were also included. No limit was applied to the databases in terms of article language. Studies that focused on participants with mental health problems (including depression, stress disorder, psychosis, and schizophrenia), or those who may have suffered from complex psychological issues often associated with serious illnesses (such as HIV/AIDS), were excluded because of their potential to weaken the study population’s representativeness and affect the generalizability of the results.

Two reviewers (one with a medical and public health background [BS] and another one with health technology and informatics background [ZSYW]) independently carried out title and abstract reviews in the screening phase. Both were well familiar with the aforementioned inclusion and exclusion criteria and evaluated the full texts of articles independently. Their results were compared, and Cohen κ was measured in the assessment of eligibility stage in order to evaluate the inter-rater reliability between the reviewers. For conflicting decisions, a consensus was arrived at between reviewers by discussing rationales and concerns and reexamining how each compromised decision satisfied the principles of inclusion and exclusion as outlined. The Rayyan web application [25] was used to facilitate the double-blind evaluations and to maintain review records. We developed a data extraction sheet for the studies included (Multimedia Appendix 2) that complied with the minimum standards of the Data Extraction Template for Included Studies, as developed by the Cochrane Consumers and Communication Review Group [26].

The details recorded (wherever available) for each included paper were as follows: general review information, study population, study characteristics, outcome measures, and results. We reported the primary outcomes and all the available secondary ones. Both qualitative and quantitative materials were extracted. We were interested in the frequency of the measurement outcomes of the intervention group (health technology) when compared with control (usual care practice). Risk difference, which is one of the most useful ways to present RCT research results, was used as a quantitative reporting measure. The outcome measures with the largest possible intervention timeframe for results were reported. The study protocol was published in the PROSPERO registry on August 7, 2018 (Registration number: CRD42018096627).

### Outcome Measures

Medication adherence [27] refers to the degree to which patients’ medication-taking behavior accords with appropriate medical advice [28]; it was set as the primary outcome measure in this study. For the secondary outcome measures, we included indications of adverse event (or safety outcome), quality of life, patient satisfaction, and health expenditure/spending, as eHealth studies often assess these health care measures and considering them would allow us to evaluate eHealth impact more comprehensively.

### Health Technology Functions

It is common for eHealth applications to have multiple features. Referring to [4] and the range of capabilities of drug application [21], we compiled a list of commonly used health technology functions, namely, mechanisms to communicate with caregivers, monitoring health features, reminding and alerting, providing information and education, dispensing treatment and administration support, personalized feedback, reporting and trending, dynamic treatment adjustment, social support, and setting goals and planning. Based on the eHealth intervention described in each study included, we tallied the occurrence of these functions to determine how often the functions were applied in eHealth interventions. Considering the presence of each health technology function and improved medication adherence at *P*<.1, *P*<.01, and *P*<.001 as variables, we constructed 2 × 2 contingency tables to examine whether the proportions for different health technology functions were different. Fisher exact test of independence was employed.

### Assessment of Methodological Quality

Two reviewers (BS and ZSYW) also performed quality assessment for the studies included using the Cochrane Collaboration’s Risk of Bias (RoB) assessment tool via RevMan version 5.3 software [29]. Following the RoB assessment guidelines [30], the reviewers assessed the RoB signaling questions as either *Low risk*, *High risk*, or *Unclear* RoB, based on the evidence accessible from each of the studies included. A third-party opinion was sought from another coauthor (KDSL), as and when needed.

## Results

### Literature Search

**Figure 1:**
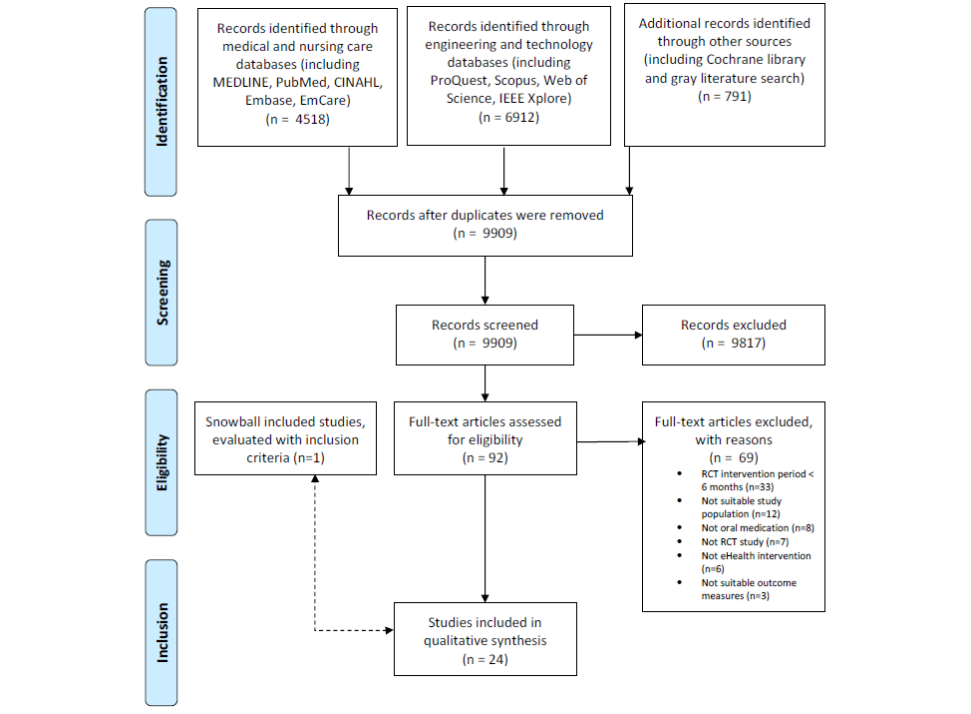
Flow diagram. RCT: randomized controlled study.

Figure 1 presents the flow diagram of the literature search (JMIR hand-searched result is appended in Multimedia Appendix 3). After removing duplicates, our initial search yielded 9909 records, of which 92 were reviewed for full-text assessment and 24 satisfied the inclusion criteria and were included in this review. Cohen κ between the reviewers was 0.846, which is equivalent to a strong level of agreement.

Table 1 illustrates the characteristics of the studies included. All the studies included were published after 2008. Of the 24 studies included, 11 (46%) [2,31-40] were published in the last 5 years (since 2014) and 20 (83%) [2,3,31-48] were published in the last 10 years (since 2009). All the studies included were published in English. As many as 10 [2,3,33-35,39,42,45,46,49] out of 24 studies were undertaken in the United States and 8 [31,36,40,41,47,50-52] were carried out in European countries. Other countries studied included Canada [43,44], South Africa [32], Iran [37], South Korea [38], and Taiwan [48]. Further, 19 [2,3,31,32,34,35,37-41,43-46,48,49,51,52] studies focused on the adult population, and only 3 [33,42,47] specifically targeted older patients (aged 65 years and older). All the health technology interventions were primarily designed for home-based usage. The relevant clinical conditions covered were asthma [41,48,50], heart disease [31,34,39,40,43,45,47,49], hypertension [32,35,37,44,46,51,52], diabetes mellitus [38], post-transplantation (renal [36] and lung [2]), comorbidities of diabetes mellitus and hypertension [3], and other unspecified diseases [33,42]. Eight studies [2,3,32,38,41,43,45,46] reported incorporation of clinical decision support or advanced computational algorithms to aid the self-management of diseases, while five [2,3,32,43,45] offered technology-led (instead of caregiver-led) dynamic feedback tailored to suit the patients’ conditions.

**Table 1:** Study characteristics.

Author, year of publication	Description of health technology intervention (intervention group size)
Jerant et al. 2003 [49]	Video-based Telecare: Aviva personal telecare unit installed at home that allows real-time videoconferencing with nurse caregiver, equipped with electronic stethoscope for lung auscultation (N=13).
DeVito Dabbs et al. 2016 [2]	Pocket PATH with a smartphone platform: Custom programs allow patient input of daily measurements (spirometry, vital signs, symptoms). Also includes decision-support feature that automatically sends reminders to patient, and to call the transplant coordinator, whenever measures reached immediate report level (N=99).
Hashimoto et al. 2011 [41]	Internet-based management tool: Included an electronic diary and treatment decision support (dose adjustment of oral corticosteroids) for patients (N=51).
Marek et al. 2013 [42]	MD.2: Medication-dispensing machine that stores and preloaded 60 plastic reusable cups in a locked compartment. Generate online compliance reports to monitor missed doses. Nurse care coordinated with physician(s) and pharmacist(s), visiting the participants at least every two weeks and performing care plan activities (N=152).
Willems et al. 2008 [50]	Electronic asthma monitor: Portable handheld device with a matching modem that can register lung function values and symptoms on the monitor (N=55).
Boyne et al. 2014 [31]	Health Buddy (telemonitoring device): Equipped with a liquid crystal display and 4 keys connected to a landline phone. Patients received daily preset dialogues and questions about their symptoms, knowledge, and behavior, which had to be answered by touching the keys. Subsequently the answers were transmitted to the nurses’ desktop (N=197).
Sherrard et al. 2009 [43]	Interactive voice response (IVR): Developed an algorithm of 11 questions addressing medication compliance, reporting of adverse events, providing information on common medications, and offering general medication safety tips. The IVR system recorded patients’ voiced responses (yes or no) into a central database (N=164).
Bobrow et al. 2016 [32]	Personalized short messaging service text messages were sent to (1) information-only message (N=457) and (2) interactive message (N=458) group participants at weekly intervals, at a time and in a language selected by the participant. Messages focused on the techniques of goals and planning, repetition and substitution, social support, and natural consequences.
Marek et al. 2014 [33]	Medication-dispensing machine + nurse care coordination (every 2 weeks), preloaded with medications in reusable plastic cups. (N=150).
Volpp et al. 2017 [34]	Vitality GlowCaps: 4 electronic pill bottles used for cardiovascular medications (including β-blockers, statins, aspirin, antiplatelet agents), which electronically monitored openings. Transmitted information to health organizations (N=682).
Kim et al. 2016 [35]	Withings Blood Pressure Monitor with iPhone with apps: Provided portals and a dashboard to link with families, caregivers, and health care professionals. Equipped with an online disease management program featuring educational materials (N=52).
Rinfret et al. 2009 [44]	IT-supported program: Consisted of educational booklet, digital home blood pressure monitor, logbook, and access to a telephone-linked management program. The system collected self-recorded blood pressure and self-assessed adherence data and integrated these data with actual pharmacy medication refill. Able to generate reports (N=111).
Santschi et al. 2008 [51]	Participants received drug with electronic monitoring devices: Medication Event Monitoring System (MEMS, AARDEX Ltd) used to obtain accurate, detailed dynamic, and *real-time* information on the patients’ medication-taking behavior (N=34).
Stacy et al. 2009 [45]	IVR system: Provided three separate tailored behavioral support interactions, coupled with tailored feedback based on parents’ cholesterol-related knowledge, attitudes, beliefs, and perceived barriers to medication adherence (N=253).
Bosworth et al. 2011 [46]	All intervention groups utilized wireless home blood pressure monitor (automatically transmitted) and telemedicine device—connected to a telephone line like an answering machine. (1) Behavioral management—nurse-administered encounter via software platform to provide health behavior modules focusing on hypertension self-management improvement (N=147); (2) medication management—triggers sent to physician and nurse to adjust medication dynamically with decision support, with nurse follow-up call every 3 weeks (N=149); (3) a combination of A and B (N=147).
Dusing et al. 2009 [52]	A set of medication supportive measures: Offered support to both physician and patient. Patient received 24-hour timer, reminding stickers, information brochures, and home blood measurement device. Electronic MEMS utilized (N=97).
Henriksson et al. 2015 [36]	Electronic Monitoring Drug Dispensing Device: The patients loaded the device with a week’s worth of medication at a time. The device generated visual and audible signals. If the patient did not take their medication, the audible signal repeated with increasing frequency for 120 minutes. After this (or after the medication was taken), the device sent an SMS text message to the web-based software, thus registering patient compliance information (N=40).
Hosseininasab et al. 2014 [37]	Wrist self-monitoring device: A blood pressure measurement device with log-book documentation (N=97).
Jeong et al. 2018 [38]	Patient in all groups used a Smart Care Unit (SCU), which consists of a web-enabled computer with camera (for videoconferencing and communication with caregiver), specific software, glucometer (blood glucose monitoring), and body composition organizer (for body weight measure, tracking diet, and exercise record). Other functions included automated short message feedback and access to care center education program. (1) Telemonitoring group: face-to-face outpatient hospital visit scheduled with caregiver at 8, 16, and 24 weeks. Medication was prescribed based on SCU data and caregiver received advice from clinical decision-support system (N=113); (2) Telemedicine group: in weeks 8 and 16, patients contacted physicians via the SCU, and in week 24, a face-to-face visit was scheduled (N=112).
Kooy et al. 2013 [47]	Electronic reminder device (ERD): Medication reminder device that beeped every day at the same time until the patient switched it off. Patients could adjust the beeping time. (1) Counseling with an ERD (N=130). (2) ERD with written instructions (N=123).
Liu et al. 2011 [48]	Mobile telephone-based interactive self-care system: Provided an electronic diary to record patients’ daily asthma symptom score (including sleep quality, coughing severity, difficulty breathing, and daily activities affected by asthma), use of relievers, peak expiratory flow rate (PEFR), and PEFR variability (N=43).
Wakefield et al. 2012 [3]	Home telemonitoring device: Employed standard telephone line to transmit data between patient and study center. Patients in all groups manually entered blood pressure and blood glucose measures. (1) High-intensity group received health information tips and questions from the branching algorithm (N=93). (2) Low-intensity group: Did not receive the informational tips and questions from the algorithm (N=102).
Young et al. 2016 [39]	One-on-one in-hospital self-management training + telephone-based postdischarge reinforcement sessions—scheduled twice a week in the first 2 weeks, once a week in weeks 3-6, and every other week in weeks 7-12. Intervention content presented in verbal, written, visual formats with interactive ability; self-management workbooks and self-management toolkit (calendar for weight and salt daily logging), weight scale with large and bright readings, and an electronic pill organizer reminder alarm are provided. Session lasted for 45-50 minutes. Booster sessions were delivered to those struggling with self-management at home. Tailored intervention sessions were provided based on level of activation, predefined goals, and specific self-management needs (N=51).
Wald et al. 2014 [40]	SMS text messaging group: Sent daily texts for 2 weeks, and alternate-day texts for 2 weeks. Subsequently sent weekly texts for 22 weeks (6 months in all). Participants were requested to reply to each message to indicate if they had taken their medication or not and if the message reminded them to take medication. Computer sends the text message based on the schedule. Patients responses were filed and if not taking medicine, telephone follow-up was made (N=151).

### Outcome Measures: Medication Adherence

Table 2 presents the definitions of different medication adherence measures and summarizes each of these measures as reported by the studies included. Among 19 studies [2,3,31,32,34-40,42-45,47,49,51,52] that evaluated and reported the impact of health technology on medication adherence, 5 [3,31,32,39,43] adopted questionnaires and scales, which are the most commonly used measures. Further, 15 [2,3,31,32,34,37-40,43-45,47,49,52] explicitly compared improvements in medication adherence as a result of the intervention with the control arms. As many as 12 [2,3,31,32,37-40,43-45,52] reported that the intervention arms had seen improvements in medication adherence at the significance level of *P*<.1. A total of 4 studies [32,38,40,43] showed significant improvement in medication adherence at the *P*<.01 level and 3 studies [32,38,43] were significant at the *P*<.001 level. These results indicate that eHealth can improve patients’ medication adherence in nonhospital settings. Multimedia Appendix 2 provides details of the synthesized outcomes, risk differences, and *P* values between intervention and control.

### Outcome Measures: Health Care Outcome Measures

Table 2 also presents the definitions of the secondary outcome measures and summarizes each of them as reported by the studies included. In all, 11 studies [32-34,38,41-43,46,48-50] reported secondary outcome measures including those that are associated with adverse events [38,43], quality of life [41,42,48,50], patient satisfaction [32,41,43,49], and health expenditure [33,34,46]. Among the 2 studies [38,43] reporting adverse events relating to the interventions (or safety outcome), no statistically meaningful difference between the intervention and control was found. Two [48,50] of the 4 studies reporting quality of life of the patients [41,42,48,50] revealed that there were significant differences between the intervention and control arms at the significance level of *P*<.01. Among the 4 studies measuring patient satisfaction [32,41,43,49], 1 [41] observed a difference when compared with the control. Three studies (all conducted in the United States) [34,42,46] reported medical spending obtained from various sources, including insurance claims [33,34] and inpatient and outpatient costs [46], and none of these showed any significant difference between intervention and control. The above evidence indicates that eHealth for self-administration of medication can improve the quality of life of patients.

**Table 2:** Summary of outcome measures.

Outcome measures			Definition	Reference(s)	
**Medication adherence (N=19)**					
	Continuous, multiple-interval measures of medication acquisition (CMA)	CMA is the sum of days of medication supply obtained divided by the total number of days of study participation [27,53,54].			[44]
	Proportion of days medication covered (PDC)	PDC measures the persistence to the medication therapy by calculating the total days’ supply divided by the number of days of study participation. The value is capped at 100% [27]. It is a common proxy-measure of adherence.			[32,34,45,47]
	Continuous measure of medication gaps	This measure refers to the total number of treatment gap days divided by the duration of the time period of interest. It indicates the proportion of time for which patients do not have drug exposure [53].			[44]
	Medication possession ratio (MPR)	MPR is the proportion of days’ medication supply obtained over either refill interval or fixed refill [53]. MPR is calculated for the individual patient and can create different denominators.			[45]
	Eight-item Morisky Medication Adherence Scale (MMAS-8)	MMAS-8 is a validated medication adherence scale that contains 7 Yes/No responses and a 5-point Likert response [53]. The scale captures patients’ medication-taking behavior and barriers to adherence.			[35,37]
	Pill count	Pill count is the number of consumed pills divided by the number of total prescribed pills [53].			[37]
	Measurement cutoff	This measure requires setting an arbitrary cutoff value to a continuous measure for identifying adherence and nonadherence into dichotomous outcomes [53,55]. It is typically less sensitive than the original measure [55]. For instance, taking medication with 80% cutoff of a 28-day medication cycle, or PDC with 80% or above.			[2,32,40,49]
	Measures involving electronic medication devices	Electronic medication devices aim to record adherence performance for analysis. Typical features include recording dosing events, audio/visual reminders, electronic displays, and monitoring and feedback on adherence performance. However, not all features are available in all devices. In many medication adherence studies, the MEMS is commonly used [53].			[36,40,42,51,52]
	Other questionnaires and scales	These questionnaires and scales are generally validated against other conditions and related measures to assess medication regime conditions for a broad range of diseased populations [53] or specific ones. Heart failure compliance scale and validated diabetes mellitus regimen adherence scale (Edwards Scale) are some examples. Self-report questionnaires are also included.			[3,31,32,39,43]
	Unspecified	Medication adherence measure was not specified.			[38]
**Health care outcome measures (N=11)**					
	Adverse event	Measures refer to the number of emergency visits or instances of hospitalization or untoward medical occurrence.			[38,43]
	Quality of life	Various measures may apply, including Short Form-36/Short Form-12 Physical Component Scale and Mental Component Scale, Asthma-Related Quality of Life, and (Pediatric) Asthma Quality of Life Questionnaire.			[41,42,48,50]
	Patient satisfaction	This measure refers to how well eHealth met patient expectations. Various measures may apply, including the 8-item Client Satisfaction Questionnaire and the patient satisfaction survey.			[32,41,43,49]
	Health spending	Various measures may apply, including assessing monthly or yearly expenditure via claims data, and medical cost computed by direct and indirect outpatient and inpatient cost items.			[33,34,46]

### Health Technology Functions

**Figure 2:**
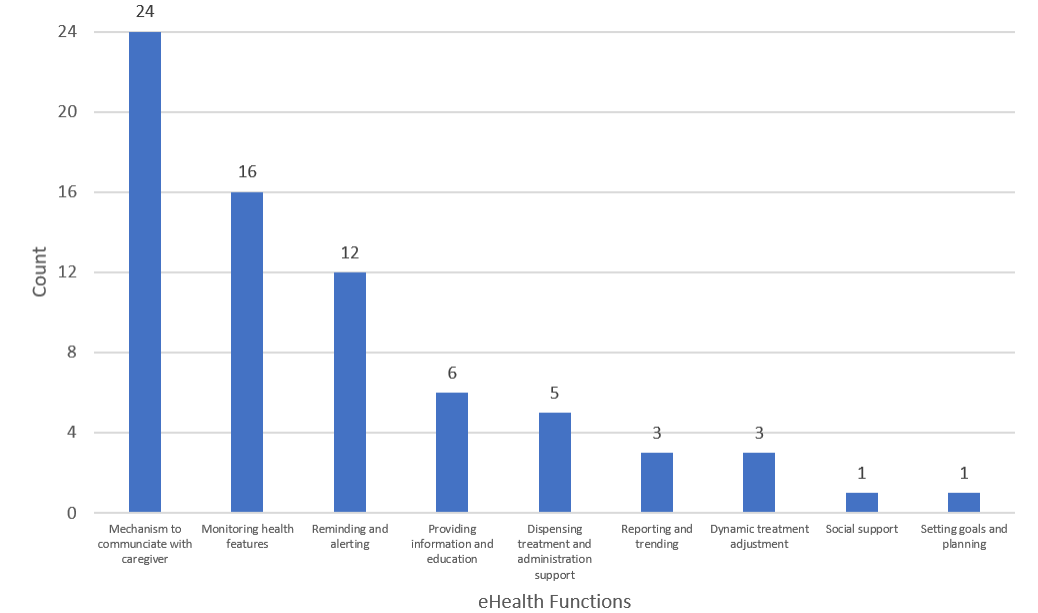
Functionalities among the studies included.

Figure 2 presents the functionalities in the studies included by inclusion frequency. It appears that all the studies included incorporated an established mechanism to communicate with caregivers. Monitoring health features [2,3,35-39,41,43,44,46,48-52], and reminding and alerting [2,31,33,35,36,39,40,42,46,47,51,52] are the second- and third-most commonly used functions, respectively. Of the 24 studies, 6 [3,32,35,38,43,52] used the function of providing information and education to users and five studies [33,34,36,39,51] incorporated dispensing treatment and administration support, such as pill organizer automatic opening devices [34]. Providing information and education and dispensing treatment and administration support are significantly different in the medication adherence (at *P*<.001 level) group (Fisher exact test, *P*=.02). This indicates that these health technology functions are effective in improving medication adherence (Details provided in Multimedia Appendix 4).

### Assessment of Methodological Quality

**Figure 3:**
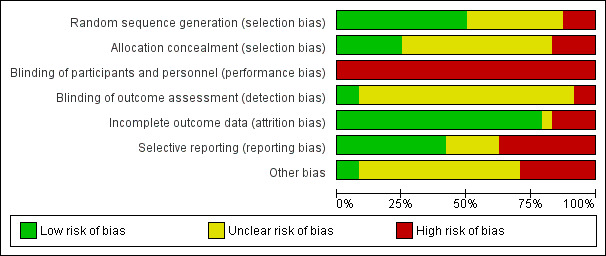
Summary of the quality of the studies included.

**Figure 4:**
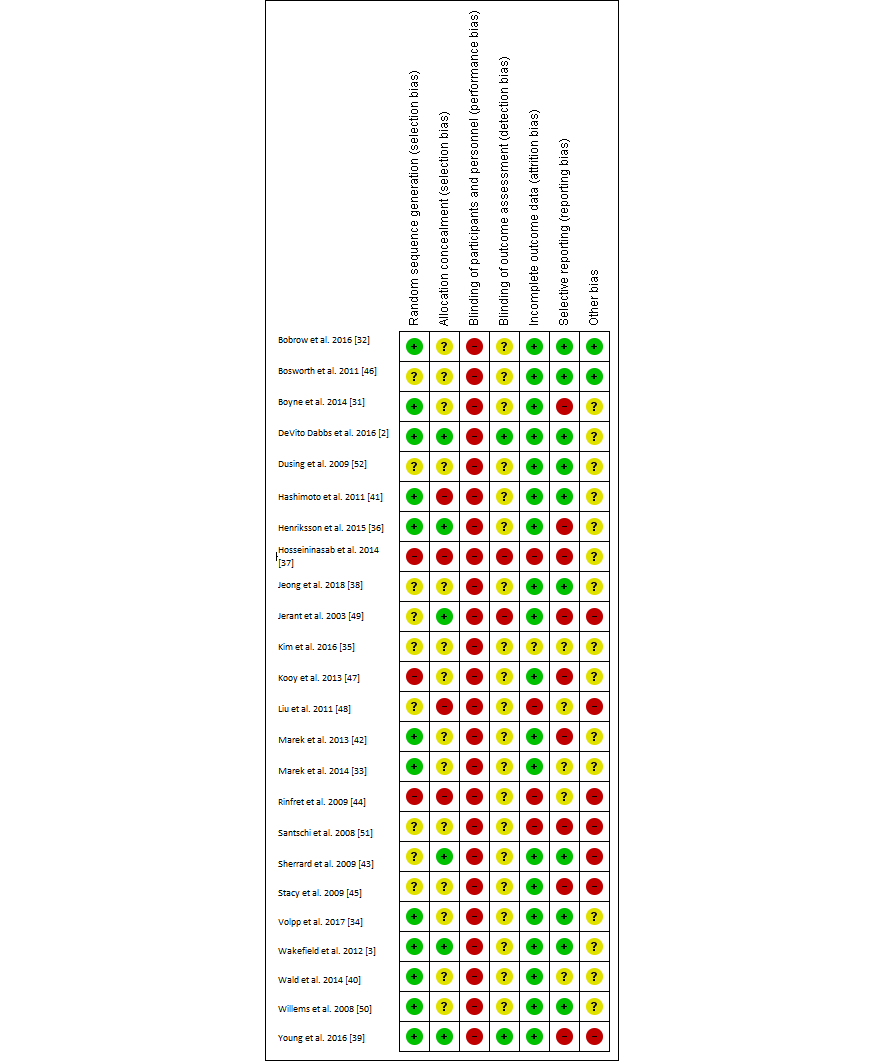
Risk of Bias (RoB) assessment of the studies included.

Figures 3 and 4 present a summary of the RoB assessment and judgments of each assessment domain for the studies included. Of the 24 studies, 12 [2,3,31-34,36,39-42,50] reported on the employment of random sequence generation methods that sufficiently produced comparable study arms, while 6 [2,3,36,39,43,49] utilized methods to conceal the allocation sequences before or during enrollment. These studies exhibited a low risk of selection bias. All the studies included had a high risk of performance bias, which refers to the lack of blinding of participants. It was found that some studies [2,32,34,37] managed to introduce blinding to the investigator or personnel engaged in the research. In terms of detection bias, only 2 studies [2,39] were classified as having low bias with an indication of postallocation assessors blinding and 20 studies were assessed as unclear due to unspecified information. The attrition rates were generally low among the studies and only 4 [37,44,45,48] were grouped as high risk in this domain. A total of 9 studies had high selective reporting bias owing to the limitation of the chosen outcome measures [31,39,45,49] or the design [37,42,47], or the inability to measure the control group’s outcomes [36,51], etc. Apart from the above, there were a large number of studies that did not provide sufficient details on how to handle various domains of RoB in the publications and that inevitably increased the challenges involved in assessing various biases in the RCT studies included.

## Discussion

This systematic review synthesized the existing evidence on the impact of eHealth interventions on medication adherence and selected health care outcome measures in nonhospital settings. There is evidence proving that eHealth improves patients’ medication adherence and quality of life. The most frequently used functions are the mechanism of communication with caregivers, monitoring of health features, and reminding and alerting. Further, providing information and education, and dispensing treatment and administration support are most favorable when it comes to improved medication adherence outcomes.

### Outcome Measures

All the studies included were designed for patient home-based care applications and chronic conditions. Chronic disease management and home health care are important health issues in the context of a rapidly aging population. Only 3 [33,42,47] of the studies included were targeted at older patients aged 65 or above, and their findings were either insignificant [33,47] or inconclusive (no control group outcome was measured) [42]. Furthermore, the conclusions drawn from interventions in the adult population may lack generalizability in explaining the impact upon an older population. This is due to potential divergence in acceptance of technology and ability to use technology-assisted services between the older population and the general adult population. To address this issue, future studies may consider specifically designing and evaluating patient-centered eHealth interventions that cater to the special needs of older patients.

All the studies integrated more than 2 functions into the health technology intervention, indicating that multimodal intervention tends to be a commonly adopted model for eHealth design. We found that studies with significant improvements in medication adherence were all equipped with 3 or more integrated functions, except [37]. For instance, Jeong et al. [38] presented a multifunctional eHealth intervention comprising heterogeneous functions such as web-enabled videoconferencing that helped connect with the caregiver, a blood glucose monitoring device, a body composition organizer, an automated short message feedback system, and access to care center education program, which achieved significant medication adherence improvement (*P*<.001). This finding was in line with the non-eHealth medication nonadherence studies that showed that multiple components incorporated into the intervention tend to be successful [6]. This may be because medication nonadherence is usually multifactorial [6,53] and is a complex behavioral issue that involves socioeconomic and therapy-, patient-, condition-, and health system/health care team-related factors [56]. Single-function interventions thus tend to be insufficient when it comes to tackling such a complex problem

All the studies included provided a common eHealth function, that is, a mechanism to communicate with the caregivers. This allowed regular patient status updates, sending alerts, remote coaching, and interactive treatment plan adjustment. Considering the sociotechnical aspect of health informatics applications, future studies should carefully consider the interplay between the health system and eHealth interventions to tackle patient self-management of diseases effectively.

Our results indicated that functions of providing information and education, and dispensing treatment and administration support tend to favor improved medication adherence outcomes. This may be because dispensing treatment and administration support offer a mechanism to regulate and track medication activities [57], and to support a smooth self-medication process. It is also important to educate patients so that they understand the related disease characteristics and the benefits of following the medication regime, through the function of providing information and education.

### The Quality of the RCT Studies Included

The results should be interpreted with caution. Many studies that we included did not explicitly report RoB assessment items, particularly in the domains of blinding of outcome assessment (83% [20/24] unclear risk) and method for allocation concealment (58% [14/24] unclear risk). This increased the difficulties in evaluating the quality of the RCTs in full in order to make comparisons and draw appropriate conclusions. DeVito Dabbs et al. [2] and Young et al. [39] are relatively high-quality RCTs that evince minimal risk in selection, detection, and attrition biases.

Owing to the disparities in population characteristics, intervention design, functionality, and reporting adherence measures, it is difficult to draw inferences and definitive conclusions on some potential hypotheses, such as the combination of eHealth functions that contribute to improvements in medication adherence for a particular disease. Evidence on the effectiveness of eHealth on medication adherence and health care outcomes improvement exists, but is not compelling enough. Larger-scale RCTs with greater sample sizes are necessary to draw inferences among those infrequently observed measures such as safety outcomes in adverse events, hospitalization, and emergency visits.

Subjective and objective measures have their pros and cons [53]. To increase measurement sensitivity, future studies can consider employing a combination of measures to assess medication adherence. When designing eHealth interventions, it is important to be aware of how the selection of outcome measures can contribute to the trustworthiness of a study. The choice of medication adherence measures can affect who is assessing the outcome and the objectivity of the assessment. In general, where subjective outcomes are concerned, blinding is particularly important. For instance, medication adherence via questionnaire survey can be subjectively assessed by the participants, which is considered as low-quality adherence measurement. Such a design can influence the blinding of outcome assessment (ie, increasing detection bias).

### Strengths and Limitations

This study attempted to examine how eHealth for patients’ medication management improves drug adherence and other health care outcome measures. Our study has a number of strengths. First, we comprehensively searched cross-sectional databases in the fields of medicine, nursing care, public health, science, engineering, and social science. We focused exclusively on studies that employed RCT, which is considered the highest standard of eHealth evaluation. Our search strategy was broad and involved a large number of studies for screening (eg, 9909). We successfully retrieved a solid body of evidence that indicated improvement in drug adherence through the application of eHealth. We also evaluated the quality of the studies included through the state-of-the-art Cochrane Collaboration tool. Our study provides practical insights into the future of eHealth design and applications for patient self-medication management.

Our study has a few limitations, as well. First, a publication bias may exist owing to the inherent tendency to publish *positive* results with significant findings. Furthermore, the studies included reported multiple outcome measures rather differently. Owing to this heterogeneity, it was not possible to carry out a meta-analysis. We synthesized review outcomes from studies that largely centered on chronic diseases. However, our search strategy was designed to consider a broad scope of studies. Based on our study framework, future studies can investigate how eHealth impacts a specific targeted disease or patient group (such as those who suffer from mental health issues or HIV/AIDS).

Furthermore, owing to the small sample size, we were unable to use more accurate parametric methods, and thus unable to ascertain the impact of a combination of multiple functions. Large-scale evaluation studies in the future can consider examining the impact of the integration of multiple functionalities into eHealth; studying the social and behavioral differences across targeted populations in reacting to health technology; and carefully evaluating other important health outcome measures including adverse events owing to health technology introduction, and cost and economic evaluation.

Understanding the nature of the eHealth intervention, one must be aware that some limitations in the study design are inevitable. For instance, all the studies included were unable to blind the intervention group from the control group (ie, the usual care group without using technology intervention). At present, there is no way for researchers to avoid performance bias in the studies reviewed. However, this situation may change in the future when successful health technology becomes the gold standard and replaces the current usual care system which does not employ health technology. Future RCTs for evaluating eHealth interventions should follow best practice guidelines, which include observing the limitations of the study design; blinding of participants, personnel, and assessors involved; selecting the most objective outcome measures; employing fair assessment methods; and avoiding selective outcome reporting.

### Conclusion

This study investigated how eHealth interventions could affect patient medication adherence and health outcomes and identified the eHealth functions that are most effective in improving medication adherence. The evidence reviewed shows that eHealth can improve patients’ medication adherence and quality of life in nonhospital settings. Integrating multiple functions into health technology tends to be effective in achieving enhanced medication adherence. eHealth functions of providing information and education, and dispensing treatment and administration favored an improved medication adherence outcome. However, the literature base remains small, diffuse, and inconclusive at this time. Medication-taking behavior may vary tremendously based on the patients’ medical conditions, the population studied, and the specific medications assessed. Many interesting potential medical–social–behavioral research hypotheses are yet to be posed and answered in the existing literature.

## Appendix

Multimedia Appendix 1 Search terms for the systematic review.

Multimedia Appendix 2 Details of the included studies.

Multimedia Appendix 3 JMIR hand-search flow diagram.

Multimedia Appendix 4 Fisher’s exact test results.

## Abbreviations

CMA: continuous, multiple-interval measures of medication acquisition
MeSH: Medical Subject Headings
PDC: proportion of days medication covered
RCT: randomized controlled trial
RoB: risk of bias
SMS: short messaging service
