# Supplementary material for: Improving Patients’ Medication Adherence and Outcomes in Nonhospital Settings Through eHealth: Systematic Review of Randomized Controlled Trials
Source: J Med Internet Res. 2020 Aug 20;22(8):e17015. doi: 10.2196/17015 (PMC7471892; doi:10.2196/17015)
Supplement: Multimedia Appendix 1 [file jmir_v22i8e17015_app1.docx]

**S1: Search terms for the systematic review**

**Search key for Pubmed*:**

((((((technolog* AND health) OR "biomedical technology"[MeSH Terms] OR health technology[Text Word] OR device* OR "telemedicine"[MeSH Terms] OR eHealth[Text Word] OR e-health OR gerontechnology [tw] OR gerotechnology [tw] OR "distance counseling"[MeSH Terms] OR etherapy[Text Word] OR etherapy[tw] OR e-care[tw] OR "self-help devices"[MeSH Terms] OR assistive technology[Text Word] OR mhealth[Text Word] OR "technology assessment, biomedical"[MeSH Terms] OR technology assessment[Text Word] OR telehealth[Text Word] OR health informatic* OR digital intervention [tw]))) AND (("nursing"[Subheading] OR "home care services"[MeSH Terms] OR home care[Text Word] OR

"community health services"[MeSH Terms] OR community health care[Text Word] OR home* OR community care OR "outpatients"[MeSH Terms] OR out patient[Text Word] OR "Ambulatory Care "[MESH] OR domiciliary care[Text Word] OR home healthcare[Text Word] OR nonhospital* OR outpatient[Text Word] OR out-patient[Text Word]))) AND ((self administration [MeSH] OR self administration[tw] OR self admin* OR self care[MeSH] OR self care[tw] OR "drug administration routes"[MeSH Terms] OR drug administration route[Text Word] OR "drug therapy" [Subheading] OR "drug therapy"[MeSH Terms] OR drug therapy[Text Word] OR "prescription drugs"[MeSH Terms] OR prescription drug[Text Word] OR "pharmaceutical preparations"[MeSH Terms] OR medication[Text Word] OR "self-management"[MeSH Terms] OR self management [Text Word] OR self-manag* OR self manage* OR self posession[Text Word] OR pharmacol* OR "drug prescriptions"[MeSH Terms] OR medication prescription[Text Word] OR "polypharmacy"[MeSH Terms] OR polypharmacy[Text Word] OR drug management[Text Word] OR

medication management[Text Word] OR medication prescrib* OR drug prescrib* OR medication dispens* OR drug dispens* OR adnimistrat* OR dispens* OR admin* OR "self-help devices"[MeSH Terms]))) AND ((((((((((randomized controlled trial [pt]) OR controlled clinical trial [pt]) OR randomized [tiab]) OR placebo [tiab]) OR clinical trials as topic [mesh: noexp]) OR randomly [tiab]) OR trial [ti])) NOT (animals [mh] NOT humans [mh])))

*The above master search key was transformed to other databases in keeping with specific databases’ search requirements.
