# Supplementary material for: Improving Patients’ Medication Adherence and Outcomes in Nonhospital Settings Through eHealth: Systematic Review of Randomized Controlled Trials
Source: J Med Internet Res. 2020 Aug 20;22(8):e17015. doi: 10.2196/17015 (PMC7471892; doi:10.2196/17015)
Supplement: Multimedia Appendix 3 [file jmir_v22i8e17015_app3.doc]

Multimedia Appendix 3: JMIR Hand-search Flow Diagram

**Screening**

**Inclusion**

**Eligibility**

**Identification**

Records screened
(n = 188)

Records excluded
(n = 182)

Full-text articles assessed for eligibility
(n = 6)

Full-text articles excluded, with reasons
(n = 6)

- RCT intervention period < 6 months (n=1)
- Not suitable study population (n=0)
- Not oral medication (n=4)
- Not RCT study (n=1)
- Not ehealth intervention (n=0)
- Not suitable outcome measures (n=0)

Studies included in qualitative synthesis
(n = 0)

Records identified through JMIR Search page (covering all JMIR publications). All hand-searched studies relied on RCT in all fields within the study period
(n = 188)
